# Nanotechnology-Based Drug Delivery Strategies to Repair the Mitochondrial Function in Neuroinflammatory and Neurodegenerative Diseases

**DOI:** 10.3390/pharmaceutics13122055

**Published:** 2021-12-01

**Authors:** Luis F. González, Lorenzo E. Bevilacqua, Rodrigo Naves

**Affiliations:** Immunology Program, Institute of Biomedical Sciences, Faculty of Medicine, Universidad de Chile, Av. Independencia 1027, Santiago 8380453, Chile; luis.fdo.gonzalez.l@gmail.com (L.F.G.); lorenzobevilacqua@ug.uchile.cl (L.E.B.)

**Keywords:** mitochondria, mitochondrial dysfunction, neuroinflammatory diseases, neurodegenerative diseases, drug delivery, nanosystems, nanovehicle, nanomedicine

## Abstract

Mitochondria are vital organelles in eukaryotic cells that control diverse physiological processes related to energy production, calcium homeostasis, the generation of reactive oxygen species, and cell death. Several studies have demonstrated that structural and functional mitochondrial disturbances are involved in the development of different neuroinflammatory (NI) and neurodegenerative (ND) diseases (NI&NDDs) such as multiple sclerosis, Alzheimer’s disease, Parkinson’s disease, Huntington’s disease, and amyotrophic lateral sclerosis. Remarkably, counteracting mitochondrial impairment by genetic or pharmacologic treatment ameliorates neurodegeneration and clinical disability in animal models of these diseases. Therefore, the development of nanosystems enabling the sustained and selective delivery of mitochondria-targeted drugs is a novel and effective strategy to tackle NI&NDDs. In this review, we outline the impact of mitochondrial dysfunction associated with unbalanced mitochondrial dynamics, altered mitophagy, oxidative stress, energy deficit, and proteinopathies in NI&NDDs. In addition, we review different strategies for selective mitochondria-specific ligand targeting and discuss novel nanomaterials, nanozymes, and drug-loaded nanosystems developed to repair mitochondrial function and their therapeutic benefits protecting against oxidative stress, restoring cell energy production, preventing cell death, inhibiting protein aggregates, and improving motor and cognitive disability in cellular and animal models of different NI&NDDs.

## 1. Introduction

Mitochondria are vital organelles in eukaryotic cells that control diverse physiologi-cal processes related to the production of energy and also cellular processes such as cell death, calcium homeostasis, and the generation and modulation of reactive oxygen spe-cies (ROS) levels [1,2,3]. Mitochondria are highly dynamic and regulated by a fine balance between biogenesis and the degradation of defective organelles [4,5]. The shape, distribution, and size of mitochondria are controlled by coordinated cycles of fission and fusion known as mitochondrial dynamics [6], whereas damaged mitochondria are selectively removed by mitophagy. Biogenesis, mitochondrial dynamics, and clearance are crucial for the functional state of mitochondria. Abnormalities or an imbalance affecting these events may have detrimental effects on mitochondria biology and cell viability [7,8]. Diverse studies performed in cell cultures, animal models and patients have shown that disturbances in mitochondrial structure and function are involved in neurodegeneration leading to motor and cognitive deficits in neuroinflammatory (NI) and neurodegenerative (ND) diseases (NI&NDDs) such as multiple sclerosis (MS), Alzheimer’s disease (AD), Parkinson’s disease (PD), Huntington’s disease (HD), and amyotrophic lateral sclerosis (ALS). Indeed, a common hallmark of different NI&NDDs is a bioenergetic deficit resulting from mitochondrial dysfunction. Furthermore, the impaired function of mitochondria increases ROS production and oxidative stress exacerbating mitochondrial damage and the progression of neurodegeneration [9,10]. In addition, structural and functional altera-tions in mitochondria are also associated with the pathological accumulation of protein aggregates in NI&NDDs. Remarkably, the restoration of mitochondrial function leads to cell damage recovery and the amelioration of clinical symptoms in cellular and animal models of NI&NDDs [11,12,13,14,15,16]. Therefore, strategies designed to restore mitochondrial homeostasis represent potential therapies for NI&NDDs and should consider not only physicochemical characteristics of drugs but also delivery formulations and biological barriers in order to reach intracellular targets in the central nervous system (CNS) and to mitigate systemic side effects. In this review, we depict the main mitochondrial alterations associ-ated with the pathogenesis of NI&NDDs and review different strategies for selective mi-tochondria-specific ligand targeting. In addition, we discuss the most recent and inno-vative nanomaterials, nanozymes, and drug-loaded nanosystems developed to counteract mitochondrial dysfunction induced by altered mitochondrial dynamics, oxidative stress, energy deficit, or protein aggregation, and their therapeutic impact on NI&NDDs.

## 2. Organization of Mitochondria

**Figure 1:**
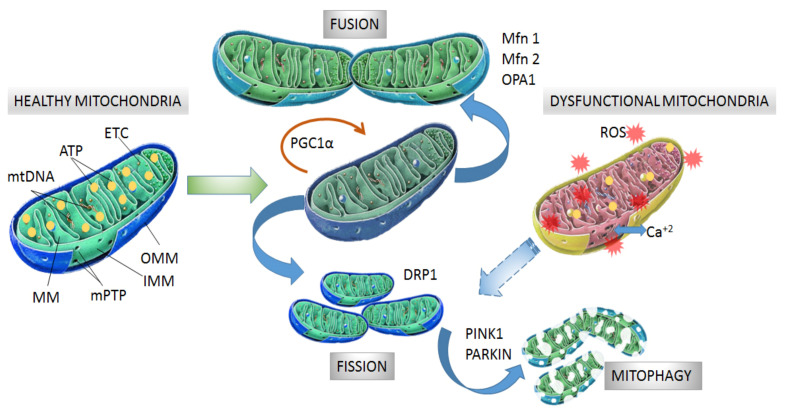
Structure and processes involved in dynamics of healthy and dysfunctional mitochondria. Healthy mitochondria display coordinated and dynamic processes of fusion and fission in order to regulate their morphology, size, and number. After mitochondrial biogenesis guided by PGC-1α protein, fusion generates an interconnected mitochondrial network, which is orchestrated by OPA1, Mfn1 and Mfn2 proteins. Fission results in small size mitochondria without mtDNA replication due to fragmentation and separation from the mitochondrial network, which is a process driven by dynamin- related protein (DRP1). Fragmented mitochondria are degraded by mitophagy, which is a process involving PINK1 and PARKIN proteins. Dysfunctional mitochondria showing alterations in structure and function in neurodegeneration are degraded by mitophagy. Mitochondrial dynamics are maintained by constant activity and precise balance between the biogenesis and clearance of fragmented and defective organelles. mtDNA: mitochondrial DNA, ATP: adenosine triphosphate, ETC: electron transport chain, MM: mitochondrial matrix, mPTP: permeability transition pore, OMM: outer mitochondrial membrane, IMM: inner mitochondrial membrane, PGC-1α: peroxisome proliferator activated receptor-gamma coactivator 1-alpha, Mfn1 and Mfn2: mitofusins 1 and 2, OPA1: optical atrophy 1 protein, DRP1: dynamin related protein, PINK1: PTEN-induced kinase 1, PARKIN: Parkin RBR E3 ubiquitin-protein ligase.

Mitochondria consist of a double membrane with an intermembrane space and an internal mitochondrial matrix (MM) that contains the mitochondrial DNA (mtDNA). The outer mitochondrial membrane (OMM) contains the voltage-dependent anion channel (VDAC) and the permeability transition pore (mPTP) associated with the unspecific trans-location of small molecules (1-5 kDa) through passive diffusion [17,18]. The inner mito-chondrial membrane (IMM) is organized in folds or cristae and contains the electron transport chain (ETC) where the oxidative phosphorylation (OXPHOS) of adenosine di-phosphate (ADP) proceeds (Figure 1). The mtDNA, located in the MM, encodes the protein subunits of respiratory chain complexes I, III, IV, and V, along with RNA components for mitochondrial protein synthesis [19]. Complex II is encoded entirely by autosomal genes [20]. These ETC complexes catalyze redox reactions from reduced dinucleotide do-nors to molecular O_2_, generating a mitochondrial membrane potential (MMP) along the IMM by pumping protons from the MM to the intermembrane space. Finally, the return of protons into the MM through an ATP synthase present in IMM drives ATP synthesis from ADP and inorganic phosphate [21,22].

Since ETC operates under the presence of O_2_, the process involves one-electron redox reactions, which demonstrates that mitochondria are the major intracellular source of ROS under normal physiological conditions [23,24,25,26]. Indeed, mitochondria generate almost 90% of cellular ROS [3]. Under pathological conditions, altered ETC function causes an exacerbation of mitochondrial ROS production leading to bioenergetic impairment and cellular and tissue damage by oxidative stress; contributing to a progressive ND process [27,28].

A relevant aspect of mitochondrial biology is the exquisite regulation of calcium con-tent. Excessive levels of mitochondrial Ca^+2^ causes increased ROS production, mPTP open-ing and impairment of energetic function [1,29,30]. In addition, the disruption of mitochondrial contacts with membranes of the endoplasmic reticulum (ER) is a crucial event for mitochondrial integrity since function and structure are highly dependent on the flux of Ca^+2^ from and into the ER [31,32].

## 3. Mitochondrial Dynamics

Mitochondrial biogenesis is controlled by the transcriptional factor peroxisome proliferator activated receptor-gamma coactivator 1-alpha (PGC-1α) which is activated by di-rect interaction with NAD^+^-dependent deacetylase sirtuin 1 (SIRT1) [33] and phosphorylation by tAMP-activated protein kinase (AMPK) [34]. Then, phosphorylated PGC-1α is translocated into the nucleus where it promotes expression of the nuclear respiratory factors (NRFs) needed for further gene expression of mitochondrial proteins [35]. Among the mitochondrial proteins expressed are ETC protein subunits, fatty acid β-oxidation proteins and mitochondrial transcription factor A (mtTFA), which drive the transcription and replication of mtDNA. Additionally, NRFs also bind to promoter regions of genes coding for ROS scavengers [36,37].

The morphology, size and number of mitochondria are regulated by coordinated cycles of fusion and fission known as mitochondrial dynamics [38]. Mitochondrial fusion generates an interconnected mitochondrial network for the exchange of matrix contents and mtDNA molecules from healthy mitochondria donors to damaged mitochondria in order to reduce altered mtDNA. The main proteins with GTPase activity involved in mitochondrial fusion are optical atrophy 1 protein (OPA1), and mitofusins (Mfn1 and Mfn2) [39,40]. Mitochondrial fission generates smaller mitochondria without mtDNA replication by fragmentation and separation from the mitochondrial network followed by processing in the autophagosome [41]. Fission is modulated by GTPase dynamin related protein (DRP1), which is recruited from the cytosol to the OMM and interacts with fission 1 protein (Fis1) to stimulate mitochondrial fission [38,39] (Figure 1).

After mitochondrial fragmentation, the removal of altered mitochondria occurs by mitophagy, which is modulated by PTEN-induced kinase 1 (PINK1) and PARKIN RBR E3 ubiquitin-protein ligase (PARKIN) proteins [42,43]. PINK1 accumulates in the OMM in response to a reduction in MMP in dysfunctional mitochondria. Then, PARKIN is re-cruited from the cytosol to the OMM and promotes ubiquitination of mitochondrial proteins, leading to mitochondrial degradation [44,45]. Recently, it was observed that PINK1 accumulation and PARKIN recruitment are required to start mitophagy which is induced by slight oscillations in mitochondrial Ca^+2^ levels in human neuroblastoma SH-SY5Y cells [46]. In addition, PINK1 is also able to activate mitophagy directly without the participation of PARKIN by recruiting optineurin (OPTN) and nuclear dot protein 52 kDa (NDP52) [47]. In this case, PARKIN participates in further amplification of mitophagy in-duced by PINK1 [11] (Figure 1).

## 4. Mitochondrial Alterations Associated with NI&NDDs

Fragmented mitochondria with altered membrane structure have been found in the brain of patients with AD, which is a neurodegenerative disorder characterized by memory and learning impairment [48]. The dysregulation of mitochondrial Ca^+2^ content and accumulation of deformed mitochondria have been reported in animal models of HD [12,49,50], which is a dominant heritable pathology characterized by cognitive impairment, chorea, dystonia, and progressive loss of motor coordination [51]. mtDNA mutations affecting the ETC complexes have been reported in patients with MS [52], which is an autoimmune disease of the central nervous system (CNS) characterized by neuroinflammation, demyelination, and axonal damage [53]. In addition, several single nucleotide polymorphisms of mtDNA have been correlated with an increased risk of MS [54,55]. In comparison to healthy individuals, higher rates of mtDNA deletions were observed in the substantia nigra of autopsied brain samples from patients with PD [56], which is a neurodegenerative disease characterized by loss of dopaminergic neurons, leading to cognitive and motor alteration. Even though precise mechanisms determining how defective mitochondria promote the ND process are elusive, recent evidence has involved exacerbated mitochondrial ROS production in cellular toxicity and the promotion of aggregation and accumulation of toxic intracellular proteins. In turn, the accumulation of toxic proteins interferes with mitochondrial function, impairing energy production and maintaining an oxidative cellular unbalance that impacts the structure and function of the CNS [57,58,59,60]. In addition, the cytosolic release of mtDNA can induce the activation of the inflammatory response [61], leading to injury and functional impairment of the CNS. mtDNA from dysfunctional mitochondria also induces the activation of nucleotide-binding domain and leucine-rich repeat (NLR) pyrin domain containing 3 (NLRP3) inflammasome proteins, which is involved in cellular apoptosis [62]. Alternatively, the cytoplasmic release of proteins from dysfunctional mitochondria such as cytochrome c is able to promote cell loss in the CNS by apoptotic mechanisms [63,64]. Interestingly, a decreased anterograde and retrograde mitochondrial transport [65,66] has been involved in the subsequent structural alterations of axons and further morphological changes of mitochondria within the spinal cord of mice developing experimental autoimmune encephalomyelitis (EAE), an animal model of MS [65]. Thus, the impaired transport of mitochondria in neurons would limit the energetic supply needed to counteract demyelination and degenerative processes in the axonal terminal. Importantly, increasing mitochondrial transport from the neuronal cell body to the axon resulted in effective protection of demyelinated axons from degeneration [67].

### 4.1. Proteinopathies and Alteration of Mitochondrial Biology in NI&NDDs

Mitochondrial dysfunction associated with NI&NDDs is also facilitated by pathological accumulation of specific aberrant proteins as a result of nuclear gene mutations or abnormal protein processing leading to oligomeric and fibrillary aggregates. The anomalous accumulation of protein aggregates impacts mitochondrial structure and function either due to altered interaction with other subcellular organelles or dysregulation of processes involved in mitochondrial dynamics. The main protein aggregates related to proteinopathies are: amyloid β (Aβ) peptide and Tau protein in AD [68,69,70,71]; α-synuclein (α-syn) in PD [72,73,74], transactive response DNA-binding protein of 43 kDa (TDP-43) in AD and ALS [61,75,76]; Cu, Zn-superoxide dismutase (SOD1) in ALS [77,78]; and Huntingtin protein (Htt) in HD [50,79,80]. Table 1 summarizes the evidence relating these protein aggregates with mitochondrial dysfunction in ND diseases. α-Syn is a neuronal protein associated with the release of neurotransmitters and synaptic vesicles [81], and its misfolding and aggregation in structures referred to as Lewy bodies, particularly in dopaminergic neurons, is a hallmark of PD [81,82]. α-syn interaction with the mitochondrial structure is associated with an impairment of ETC activity, decreased MMP, mPTP opening and mitochondrial swelling as well as increased levels of mitochondrial ROS and neuron cell death [73,74]. In addition, α-syn interferes with the mitochondrial contacts with ER, leading to the disruption of Ca^+2^ flux and a reduction of ATP production [83]. Additionally, α-syn association with mitochondria results in downregulation of the predominant SIRT in the mitochondria (SIRT3) [72], which is a molecule that protects mitochondrial integrity and energetic function [84]. SIRT3 reduction is also accompanied by an increased expression of the fission protein DRP1 in neural cells and brain tissue of mice expressing α-syn [73]. α-Syn is also upregulated in the neurons and glia of demyelinating lesions in the spinal cord of mice developing EAE [85,86]. Moreover, the levels of α-syn in the cerebrospinal fluid of MS patients correlates with disease disability, suggesting a participation of α-syn in demyelinating and NI pathologies [87]. The intracellular accumulation of aggregates of Aβ peptide and hyperphosphorylated protein Tau are biochemical characteristics of AD [71,88,89]. The Aβ peptide derives from amyloid precursor protein (APP) in the mitochondria–ER contacts through sequential processing by BACE1 (β-site APP cleavage enzyme 1) and γ-secretase [90]. Aβ interacts with mitochondrial molecules and structures, which results in ROS production, mitochondrial dysfunction, and subsequent cell damage [91,92,93,94]. In addition, Aβ alters mitochondrial morphology and fragmentation by increasing DRP1 and reducing Mfn1 levels in cellular models [68,94]. Moreover, the mitochondrial fission promoted by Aβ occurs through *O*-GlcNAcylation of DRP1 in both neuronal cell lines and primary cultured neurons [69]. Interestingly, the activated form of DRP1 by *O*-GlcNAcylation is also found in the brains of mice in an AD mouse model [69]. Tau is a microtubule-binding protein in neurons, and its abnormal processing and aggregation by hyperphosphorylation promotes dissociation of preformed microtubules, interaction with mitochondrial ETC complexes, reduction of ATP production, and neuronal death [71,93]. In addition, hyperphosphorylated Tau interacts with VDAC1 mitochondrial protein promoting the alteration of energetic functions, Ca^+2^ homeostasis, and oxidative balance [71,91]. In addition, Tau alters mitochondrial fission and mitophagy by interacting with DRP1 and PARKIN proteins, respectively, in both patients and transgenic mouse models of AD [70,93]. TDP-43 is an essential ribonucleoprotein that can also form toxic cytosolic aggregates in AD. Diverse mutated forms of TPD-43 have been localized as aggregates in the mitochondria of mouse models and patients with familial ALS and are associated with structural and functional alterations in mitochondria [61,95,96]. Consistently, the inhibition of mitochondrial localization of TDP-43 restored mitochondrial function and ameliorated motor and cognitive deficits in ALS and AD models [75,76,97]. In HD, the generation of a mutated huntingtin protein (mHTT) by abnormal expansion of a CAG polyglutamine trinucleotide [98] impacted mitochondrial function and subsequent ND processes [50,79,80]. The enzyme SOD1 is mainly a cytosolic molecule, but mutated forms of SOD1 have been localized in aggregates associated with mitochondria in transgenic mouse models and patients with familial ALS [77,78]. Interestingly, the pharmacological reduction of misfolded SOD1 restored the structural integrity of mitochondria, reduced degeneration of motor neurons, and attenuated motor deficits in a transgenic ALS mouse model [99].

### 4.2. Alteration of Mitochondrial Dynamics in NI&NDDs

Mitochondrial dysfunction associated with NI&NDDs is also characterized by the altered activity of key proteins involved in mitochondrial dynamics. Diverse studies performed in cellular and animal models of NI&NDDs as well as in postmortem brain tissue of patients with NI&NDDs have shown increased activity of fission proteins such as DRP1 and FIS1 and in some cases reduced levels of Mfn and OPA proteins [50,69,80,94,101,106]. Interestingly, reversing DRP1 activation by pharmacological or genetic inhibition reduced mitochondrial fission and cell death in cellular and animal models of ND diseases [107,108,109]. Alternatively, increasing the mitochondrial fusion through overexpression of Mfn2 restored mitochondrial dynamics and attenuated neural damage and motor deficits in NI&NDDs [110].

In addition, impaired mitophagy has been observed in several studies with animal models of NI&NDDs [46,79,111]. Dysfunctional mitophagy has been associated with altered aspects of PINK and PARKIN function; such as their inactivation, deficient expression, suppression of mitochondria calcium signaling hampering recruitment of PINK and PARKIN to the mitochondria [46], or gene mutations affecting these proteins. Mutations in PINK and PARKIN genes have been related to the origin of familial PD, which can represent around 10% of the diagnosed forms of PD [112,113,114]. Interestingly, normalizing PINK and PARKIN activity through chemical agents restored mitophagy and cognitive impairment in ND models [13,115]. Additionally, PINK1 overexpression rescued mitochondrial dysfunction and restored the removal of defective mitochondria in transgenic animal models of AD and HD [11,12,116].

Recently, a rat model of PD induced by direct intracerebroventricular injection of 6-hydroxydopamine (6-OHDA) exhibited accumulated defective mitochondria as spheroids in dopaminergic neurons by unfinished mitophagy. Interestingly, altered mitochondria were transferred to surrounding astrocytes in order to complete the remaining steps of mitophagy [117]. The cellular expulsion of damaged mitochondria has also been observed in a PD model of neuronal cells treated with rotenone and in fibroblasts and cerebrospinal fluid samples from PD patients carrying a PARKIN mutation [118]. A self-destructive process of mitochondrial removal referred to as mitoautophagy has been proposed to operate in the motor neurons of an ALS transgenic mouse model. Unlike classical mitophagy, mitoautophagy produces mitochondrial degradation without the participation of lysosomes or autophagasomes [105].

Decreased mitochondrial biogenesis is observed in both patients and animal models of ND diseases [119,120]. The impaired process is characterized by the altered expression of PGC-1α, which is the main molecule involved in mitochondrial biogenesis. PGC-1α expression is significantly reduced in the brain tissue of HD mice [80] as well as in postmortem brain tissue of AD [121] and MS patients [122]. Additionally, PGC-1α has been found post-translationally inactivated by phosphorylation in the neurons of spinal cords from EAE mice [14]. Consistently, mice overexpressing neuronal PGC-1α showed an increased number of active mitochondria with enhanced respiratory capacity and a significantly better recovery of clinical disability and neurodegeneration induced by EAE compared to wild-type controls [14].

### 4.3. Energy Impairment Associated with Mitochondrial Dysfunction in NI&NDDs

A common hallmark of different NI&NDDs is the bioenergetic deficit due to mitochondrial dysfunction. ETC activity and ATP production declines in mitochondria in early stages of AD and ALS [123,124] and an altered brain energy metabolism, evidenced by a reduced neuronal glucose uptake, is associated with the progression of neurodegeneration and frequently manifests before symptomatic onset in AD, PD, ALS, and HD [125,126,127,128,129]. In addition, brain glucose hypometabolism correlates with subsequent patterns of motor and cognitive deficits along with pathology progression [125,130,131]. Reduced glucose uptake in the brain of patients with ND diseases associates with brain insulin receptor desensitization, as evidenced by the significant reduction and deterioration of insulin receptors in AD [132,133]. Interestingly, insulin regulates the function of mitochondria by upregulating the ETC complex proteins [134] and brain insulin resistance has been related to mitochondrial dysfunction and promotion of PD [135]. Consistently, evidence (see Section 6) shows that compounds that promote uptake of glucose as well as insulin sensitizers restore mitochondrial function and ameliorate cognitive and motor disturbances in ND mouse models. Therefore, an energy impairment promoted by the hypometabolism of glucose and dysfunctional mitochondria contributes to ND diseases [136,137,138].

### 4.4. Oxidative Stress Associated with Mitochondrial Dysfunction in NI&NDDs

Impaired operation of ETC is observed in the mitochondria of neurodegenerative tissues leading to increased ROS production and oxidative stress. The brain is very sensitive to redox impairment due to the high content of polyunsaturated fatty acids. Therefore, the brain is prone to suffer further oxidation events due to the presence of transition metals, such as iron and copper, putting mitochondria in a circular loop of damage and leading to more defective mitochondria [9,10]. Thus, dysfunctional mitochondria can exacerbate the oxidative environment in ND diseases. In turn, oxidative conditions severely affect the machinery involved in energy production exacerbating ROS production in mitochondria. For example, the expression of mtDNA genes required for OXPHOS and ETC function are significantly reduced in postmortem brain tissue of AD and PD [121] as well as in MS patients and in motor neurons of the spinal cords of EAE mice [52,57,139,140]. In these cases, reduced mitochondrial function is accompanied by an augmented oxidative response which often precedes more severe signs of biochemical and clinical defects in memory and neurodegeneration [57,58]. Studies have determined reduced activity of the complex II enzyme succinate dehydrogenase (SDH) and a decreased complex II-III activity in the basal ganglia of HD patients along with a decrease in complex IV activity in HD striatum. 3-nitropropionic acid (NPA), an inhibitor of SDH, causes mitochondrial damage leading to an increase in electron leakage from the mitochondria, production of ROS and nitrogen species, and depletion of antioxidant defenses. Interestingly, 3-NPA in rodents causes striatal degeneration and impairment in locomotor activity, body weight, and cognitive deficits that closely mimic symptoms seen in HD [141,142]. PD induced in mice or primates by diverse compounds such as 1-methyl-4-phenyl-1, 2, 3, 6-tetrahydropyridine (MPTP), rotenone or 6-OHDA produces loss of dopaminergic neurons by inhibition of ETC in the mitochondria and exacerbated generation of ROS [143,144,145,146]. Impaired mitochondrial production of ATP in motor neurons in mouse models and patients with ALS is accompanied by massive oxidative damage prior to the manifestation of clinical symptoms or at early stages of disease [60,147,148].The oxidative stress can also induce mitochondrial dysfunction by affecting mitochondrial dynamics. Reactive nitrogen species can activate DRP1, leading to mitochondrial fragmentation and ND damage in cellular and animal models of AD and MS [148,149]. Interestingly, treatment with chemical antioxidants reverted the defective operation of mitochondrial OXPHOS in cultured fibroblasts from ALS patients [16]. Thus, counteracting the oxidative stress associated with mitochondria dysfunction could be an important therapeutic strategy for tackling ND processes.

## 5. Mitochondria-Targeted Drug Delivery Nanosystems to Repair Mitochondrial Function in NI&NDDs

Strategies for mitochondria targeted drug delivery should consider not only physicochemical characteristics of drugs but also pharmaceutical formulations and biological barriers to reach intracellular targets in order to maximize drug therapeutic efficacy and minimize side effects. Indeed, the major limitations of mitochondrial delivery are uptake in recipient cells, endosomal escape, lysosomal degradation and cytoplasmic retention by binding to other intracellular structures [150]. Nanotechnology offers effective solutions to overcome limitations produced by poor bioavailability and unspecific delivery of drug molecules. The emergence of specific nanocarriers or nanoformulations (NFs) are expected to contribute to increasing the protection against biotransformation and retention, promoting the slow and sustained release of associated cargoes in order to reduce dose and frequency of administrations, enhancing the presence of drugs in target tissue and subcellular organelle, and reducing adverse-side effects.

### 5.1. Nanosystems to Counteract the Oxidative Stress Associated with Mitochondrial Dysfunction in NI&NDDs

Considering the disruption of the redox balance associated with ND conditions, the reduction of ROS levels produced in mitochondria, through molecules with antioxidant activity, would be a useful therapeutic strategy for mitochondrial dysfunction and to cope with neurodegeneration. Specific mitochondria-targeted nanosystems containing antioxidants have been proposed for such purposes (Table 2). Cerium oxide (CeO_2_) nanoparticles able to scavenge superoxide anions, hydrogen peroxide, and peroxynitrite, are internalized by neurons and accumulate at the OMM. These CeO_2_ nanoparticles reduce levels of reactive nitrogen species, Aβ-induced mitochondrial fragmentation, and neuronal cell death in a cellular model of AD [151]. Amphiphilic liposome-like structures containing the quaternary ammonium cation dequalinium (DAQ) are preferentially localized at mitochondria due to the positive density of charge in the ammonium of DAQ that interacts with the negative membrane potential in the mitochondria [152]. Using the mitochondria targeting ability of the dequalinium (DQA) moiety, DQAsome nanocarriers associated with Pluronic F68 (P68, a non-ionic surfactant) and containing the antioxidant molecule N-acetyl cysteine (NAC) alone or in combination with deferoxamine (an iron chelator), show excellent ability to protect against oxidative stress when compared to free drugs in the SH-SY5Y human neuroblastoma cell line treated with rotenone, which is the most widely used cellular model of PD [153].

Other nanostructures may need surface modification to create a coating of targeting ligands that direct the structure to the mitochondria. Specific drug delivery to mitochondria has been propelled by triphenylphosphonium (TPP), which is a lipophilic cation widely studied as a mitochondria-targeted agent [154,155]. Diverse TPP-linked antioxidants based on natural hydroxycinnamic derivatives have shown preferential mitochondrial localization and enhanced protection against oxidative damage and mitochondrial defects compared to free molecules both in cellular and animal models as well as in ex vivo assay with samples from NI&NDDs patients [156,157,158,159,160,161]. Likewise, TPP has been conjugated to different nanostructures for specific drug delivery and recovery of mitochondrial function in ND diseases. For example, CeO_2_ nanoparticles (NPs) targeting mitochondria by conjugation to the TPP moiety produce effective mitochondria accumulation and inhibit abnormal production of mitochondrial ROS induced by Aβ in SH-SY5Y and astrocytic (U373) cell lines [162]. Another study showed that Cerium-TPP NPs reduce mitochondrial but not cytosolic ROS in SH-SY5Y cells and HeLa cells upon chemically induced oxidative stress [163]. In vivo, the intracerebral injection of Cerium-TPP NPs restores the altered mitochondrial morphology along with a reduction in glial activation and neuronal loss in a mouse model of AD [162]. In addition, intracerebral administration of Cerium-TPP NPs significantly reduces the level of lipid peroxidation and restores the impaired biosynthesis of dopamine in the CNS of a mouse model of PD induced by MPTP [163].

Another example is biodegradable polymeric NPs made of poly (L-lactic-co-glycolic acid) (PLGA) and poly (ethylene glycol) (PEG) fused to TPP (PLGA-b-PEG-TPP) and loaded with curcumin. Curcumin is a polyphenol derived from *Curcuma longa* L. with reported anti-inflammatory, antioxidant and neuroprotective activity [164], but with low bioavailability, poor stability under physiological conditions, rapid systemic elimination and limited blood-brain barrier penetration in vivo [165,166,167]. Curcumin loaded PLGA-b-PEG-TPP NPs showed mitochondrial localization and effective protection against cellular death compared to a non-targeted construct in a cellular model of AD induced by Aβ toxicity [168]. In addition, a nanosystem composed of human serum albumin NPs linked to TPP and coated with a shell of red blood cell membrane (RBC) and T807 (7-(6-nitropyridin-3-yl)-5H-pyrido[4,3-b] indole moiety has been developed [169]. The RBC coating is intended to prolong systematic retention time and reduce reticuloendothelial system (RES) uptake and immunorecognition, while the T807 moiety allows the nanocarrier to accumulate in neurons after crossing the blood-brain barrier (BBB). This nanosystem loaded with curcumin showed effective reduction of mitochondrial oxidative stress and neural death in a cellular and animal model of AD [169].

MitoApo is a mitochondria-targeted synthetic molecule derived from the antioxidant molecule Apocynin (4-hydroxy-3-methoxyacetophenone) linked to the TPP moiety [170], which has good permeability through the BBB and CNS availability [161,170]. MitoApo prevents the loss of nigrostriatal neurons and glial activation by improving mitochondrial oxidative balance and ATP levels in a mouse model of PD showing mitochondrial dysfunction [161,170]. A nanosystem containing MitoApo composed of polyanhydride NPs of sebacic acid and 1,6 bis(p-carboxyphenoxy) hexane shows significant protection in H_2_O_2_-induced injured primary cortical neurons [171] and reduces mitochondrial ROS production and cell death in a PD model of LUHMES cells treated with the oxidant 6-OHDA [171].

Other groups of NFs containing antioxidant cargoes but devoid of any mitochondriatropic moiety have also improved the impaired mitochondrial function in cellular and animal models of ND diseases. Recently, a nanosystem of polymeric micelles was made of a polymer that structurally contains a stable nitroxide radical (4-amino-2,2,6,6-tetramethylpiperidinyloxy, TEMPO) as antioxidant moiety, which is able to dissolve by protonation under acidic conditions such as the gastric environment after their oral administration [172,173]. This nanosystem restores mitochondrial mass impairment [173], reduces oxidative stress, stabilizes mitochondria structure and increases ATP levels [174] in a cellular model of PD [173] as well as reduces oxidative stress markers and significantly attenuates cognitive deficits in a transgenic AD mouse model [172]. A micellar NF composed of carnosine and α-lipoic acid, two effective antioxidant molecules counteracting mitochondrial dysfunction and oxidative damage in neural cells from ND models [175,176], normalizes the antioxidant activity in the brain of rats with PD induced by MPTP. In this study, only the combined components restore dopamine and serotonin levels in the brain of PD mice [176]. A nanosystem of gold NPs (AuNPs) loaded with α-lipoic acid was able to revert the oxidative damage induced by α-syn and 6-OHDA in SH-SY5Y cells [177]. Table 2 summarizes the above described nanosystems containing antioxidant agents to relieve mitochondrial dysfunction in models of ND diseases.

### 5.2. Nanocarriers Improve CNS Bioavailability of Plant Compounds with Antioxidative and Neuroprotective Activity

Diverse plant compounds with antioxidant, anti-inflammatory and neuroprotective properties used in cellular and animal models of neurodegeneration have been reviewed extensively elsewhere [178,179]. However, most of plant chemicals described in the following sections as bioactive agents mitigating mitochondrial dysfunction have rapid metabolism and systemic elimination [180,181] along with reduced water solubility, chemical instability [181,182], and reduced permeation across the BBB [183,184,185], which limit their bioavailability and therapeutic efficacy in the CNS. Using in vitro BBB models, researchers have observed a minimal passive permeation of plant polyphenols [186] and that the permeation through BBB is more efficient for less polar methylated forms of polyphenols [183]. Other plant polyphenols, such as quercetin (QRC), also show poor permeation (≈10%) through RBE4 and hCMEC/D3 cells, which are used as in vitro models of BBB with an incubation period of 18 h [183,187], which is associated with the activity of efflux transporters in endothelial cells of the BBB. The permeation of epicatechin and catechin through an in vitro BBB model ranges between 20 and 25% in a period of 18 h [183]. In addition, the interaction with other natural plant chemicals is able to interfere and promote further reduction in the BBB permeation of catechins and QRC [183,184]. Epigallocatechin gallate (EGCG) has a poor permeation (3%) after 30 min of incubation in a BBB model composed of endothelial cells, pericytes, and astrocytes [184]. Different phenolic metabolites resulting from the chemical transformation of plant polyphenols have low rates of permeation through in vitro BBB models based on endothelial cells and co-cultures of endothelial cells, pericytes, and astrocytes [185,188].

Although it is possible that a minimal amount of polyphenols or their derived metabolites can cross the BBB to reach the CNS and exert a biological effect, their therapeutic application would require a continuous supply of elevated doses of the drug and frequent administration. In this context, the association of bioactive natural products as phenolic compounds to nanosystems not only improves their solubility and protection against chemical transformation or inactivation but also enhances their persistence in the system, allows controlled release, and increases their therapeutic effectiveness. Novel nanomaterials serving as therapeutic agents per se or associated to active drugs counteract mitochondrial dysfunction in isolated neural cells and ND of the brain (see Section 8.1 and 8.2). Nanomaterials displaying a photothermal effect presents increased BBB permeability as evidenced in the studies described in Section 8.1. Moreover, the ability to link functional moieties to the surface of nanosystems offers other options to improve bioavailability and accumulation of cargoes in specific targets. As discussed in the following sections, modified nanosystems decorated with targeting or specific stabilizing moieties resulted in higher therapeutic effects than free cargoes in the amelioration of mitochondrial dysfunction in cellular and animal ND models. For instance, nanosystems coated with poloxamer 188 (F68), polysorbate 80 (P80 or Tween 80) or T807 have promoted an efficient permeation through endothelial cells of BBB and higher brain accumulation of drugs than uncoated nanosystems [169,189,190,191]. Similar effects have been found in NPs associated with PEG [192]. Nanosystems linked to RBC membranes (RBC) or PEG moiety have showed a reduced absorption in the RES, reduced recognition by the immune system, longer circulation times and higher levels of accumulation in their targets than unmodified systems [169,193,194].

Interestingly, nanosystems associated with ligands as transferrin and lactoferrin have been used to target specific receptors expressed in the BBB and to exploit the process of receptor-mediated endocytosis to reach the brain [195,196,197,198]. These nanosystems provide significantly higher brain bioavailability and neuroprotective effects of plant chemicals in ND brains [197,198,199] (Table 3, Section 5.3 and Section 8.1). Alternatively, nanosystems made of pure plant polyphenols without other matrix materials and referred to as nanocrystals constitute more stable systems that provide enhanced cell and mitochondrial protection compared to free drugs in cellular and animal ND models (Table 3 and Section 5.4).

### 5.3. Nanosystems Containing Antioxidative and Neuroprotective Plant Chemicals to Repair Mitochondrial Function in Neurodegeneration

The association of curcumin to nanostructured lipid carriers (NLCs) increases its plasma and brain levels compared to free curcumin. The higher brain bioavailability of curcumin produces a significant reduction in mitochondrial oxidative stress and increases ATP levels in hippocampal tissue as well as a recovery of cognitive impairment in an AD mouse model [200]. A hydrogel formed by the association of NLCs, gellan, and xanthan gums were loaded with the polyphenol resveratrol (RESV), which presents low bioavailability due to rapid biotransformation, and reaches poor CNS levels in its free presentation [201,202,203]. In comparison to a free RESV suspension, the nasal administration of this hydrogel nanosystem had higher permeation in the nasal mucosa and significantly improved memory function in an AD rat model induced by scopolamine [204].

Ferulic acid (4-hydroxy-3-methoxycinnamic acid, FA), is a phenolic compound with antioxidant and anti-inflammatory properties [205,206]. FA is mostly converted into active metabolites through systemic metabolism and has poor BBB permeability and aqueous solubility [206,207]. Nonetheless, FA is able to restore altered mitochondrial dynamics by reducing mitochondrial DRP1 expression and increasing gene and protein expression of PGC1α in a 6-OHDA lesioned rat model of PD [208]. Importantly, FA associated with solid lipid NPs (SLN) reduces exacerbated ROS levels and prevents mitochondrial dysfunction at a greater extent than free FA in a cellular model of AD [209]. A nanoemulsion composed of ethyl oleate as the oil phase and a mix of surfactants (polyethylene glycol ester of 15-hydroxystearic acid: polyoxyethylene) was used to associate Osthole (OST), which is a coumarin with potent protective properties on the mitochondrial structure and function in cellular and animal models [210,211]. However, OST has rather low-moderate BBB penetration and poor stability and bioavailability [199,212]. The treatment with the nanoemulsion containing OST displayed effective cellular protection by restoring oxidative balance as well as improvement in spatial memory ability in mice with scopolamine-induced AD [213].

Some inorganic nanosystems have been used to load natural compounds as mitochondrial therapeutic agents (Table 3). QRC is a flavonoid that restores activity of ETC complexes and ATP levels in the brains of PD and HD animal models induced by treatment with rotenone and 3-NPA, respectively [214,215]. Its pharmacokinetic properties are rather poor, with mediocre bioavailability, high instability and a short half-life [216]. QRC is mostly converted into metabolites with not fully known activity [217],. Recently, an inorganic nanosystem made of superparamagnetic Iron Oxide NPs (QT-SPION) containing QRC, significantly improved the antioxidant effect of free QRC by increasing expression levels of antioxidant enzymes, reducing oxidative marker levels, and inhibiting progression of cognitive impairment in the hippocampus of an AD rat model [218]. In addition, novel nanomaterials derived from graphene have been used as carriers of natural compounds (Table 3). Dauricine (DAU) is an isoquinoline alkaloid with neuroprotective properties associated with strong antioxidant activity, the capability of stabilizing mitochondrial membranes and increasing expression of proteins associated with mitochondrial ETC and ATP synthesis in cellular [219,220] and mouse AD models [221]. However, its short half-life, rapid metabolism and poor permeability across the BBB interferes with its therapeutic potential [222]. Recently, a nanosystem consisting of DAU associated with graphene oxide (GO) NPs was significantly superior to free drug and blank nanocarrier in counteracting the oxidative unbalance, cognitive memory deficits, and astrocyte and microglia activation generated in an AD mouse model [222].

In order to increase bioavailability of natural compounds in determined organs, other nanosystems containing plant chemicals have been associated with targeting moieties (Table 3). Puerarin (PUE), an isoflavone able to revert mitochondrial dysfunction and promote mitochondrial biogenesis [223], with poor water solubility and permeability across the BBB, and short half-life [224,225]. PUE has been loaded on GO nanosheets linked to lactoferrin to bind to the vascular endothelial receptor on the BBB and to promote brain access of the therapeutic agent. The Lactoferrin-GO-PUE nanosystem provides significantly higher brain bioavailibity of PUE, reduces oxidative stress and loss of dopamine neurons in the striatum, and restores motor dysfunction of PD mice to a significantly higher extent than free PUE or a non-targeted GO-PUE carrier [198].

A nanosystem of liposomes loaded with OST was decorated with transferrin as the target ligand to promote BBB crossing and to increase OST accumulation in the brain. In comparison to free OST, the targeted OST nanosystem shows OST accumulation in the brains of AD mice leading to the stabilization of neurons and mitochondrial structure by reducing oxidative stress, neuroinflammatory markers, and Aβ aggregation. In addition, the OST nanosystem improves cognitive function in AD mice better than free OST and the non-targeted nanosystem (OST-Lip) [199].

Genistein (GEN) is an isoflavone with antioxidant and protective effects on impaired mitochondria in PD models [226,227] and is also able to boost mitochondrial energetic function by inducing the expression of genes associated with ATP synthesis [228,229]. However, it has poor aqueous solubility, oral bioavailability and brain accumulation [230], which hinders its therapeutic effects. In a more complex approach, GEN was loaded on a double-targeted nanosystem made of SLN attached to rabies virus glycoprotein (RVG29) and TPP moieties for drug targeting to the brain and mitochondria, respectively. This nanosystem prevented neurodegeneration in the hippocampus and reversed cognitive deficits in an AD mouse model to a greater extent than non-targeted nanosystem or free GEN treatment [231]. A similar double-targeted nanosystem loaded with RESV showed neuroprotective effects in an AD mouse model [232].

**Table 3:** Nanosystems containing antioxidant and neuroprotective plant chemicals to repair mitochondrial function in models of neurodegenerative diseases.

Nano-System	Drug	Disease Model	Effect on Mitochondria and Neurodegeneration					Refs
			Oxidative Stress	Cell Viability	Inflammation	Clinical Manifestations	Other Effects	
NLCs	Curcumin	AD mouse model	↓cellular and mitochondrial oxidative stress markers	neuroprotection of hippocampal cells	↓Aβ deposition in hippocampus	↑learning and memory functions	↑brain levels of curcumin, ↑ATP levels	[200]
NLCs-Gellan/ xanthan	Resveratrol	AD mouse model	-	-	-	↑learning and memory functions	↑permeation through nasal mucosa	[204]
SLN	Ferulic acid	Cellular model of AD	↓cellular and mitochondrial oxidative stress markers	↓death by apoptosis and ↑viability of neurons	↓release of mitochondrial cytochrome c	-	↑stabilization of mitochondrial membranes	[209]
Nano-emulsion	Osthole	Cellular and mouse models of AD	↓oxidative stress markers, ↑activity of antioxidant enzymes (SOD and GSH-Px)	↓death by apoptosis and ↑viability of neurons	-	↑learning and memory functions	↑acetylcholine activity in cortex and hippocampus	[213]
SPION	Quercetin	Rat model of AD	↑expression levels of antioxidant enzymes (SOD, GSH-Px, CAT)	↓apoptotic pathways	↓expression of nitric oxide synthase and amyloid precursor protein	↑learning and memory functions	↑acetylcholine activity in hippocampus	[218]
Graphene oxide NPs	Dauricine	Cellular and mouse model of AD	↓oxidative stress markers, ↑SOD activity	↓apoptosis and ↑viability of neurons	↓ glial activation	↑learning and memory functions	↑brain-derived neurotrophic factor (BDNF)	[222]
Grapehene oxide sheets/lactoferrin	Puerarin	Cellular and mouse model of PD	↓oxidative stress markers, ↑GSH levels and SOD activity	↓dopaminergic neuron loss	-	↑cognitive and motor functions	↑permeation through a BBB model, ↑brain accumulation of Puerarin, ↑dopamine levels	[198]
Liposomes/ Transferrin	Osthole	Cellular and mouse model of AD	↓mitochondrial oxidative stress, ↓lipid oxidation, ↑SOD activity	↑viability of neurons, ↓neuron apoptotic pathway	↓inflammatory markers in brain tissue, ↓Aβ accumulation in hippocampus	↑cognitive functions	↑mitochondrial stability, ↑permeation through a BBB model, ↑brain accumulation of Osthole	[199]
SLN/RVG29/TPP	Genistein	Cellular and mouse model of AD	↓mitochondrial oxidative stress	↓neuronal apoptotic pathways, ↑cellular stability in hippocampus	↓inflammatory markers and glial activation, ↓Aβ accumulation in hippocampus	↑cognitive functions	↑localization in neuronal mitochondria, ↑permeation through a BBB model, ↑RES evasion	[231]
	Resveratrol	Cellular and mouse model of AD	↓mitochondrial oxidative stress	↓neuronal apoptotic pathways,	↓glial activation, ↓Aβ accumulation in hippocampus	↑cognitive function	↑localization in neuronal mitochondria, ↓nanoparticle uptake by macrophages, ↑permeation through an in vitro BBB model	[232]
Nanocrystal	Puerarin	Cellular and mouse model of PD	↓oxidative stress markers, ↑SOD activity and GSH levels in brain	↑dopaminergic neuronal viability	-	↑cognitive and motor functions	↑mitochondrial stabilization, ↑brain accumulation of Puerarin, ↑dopamine levels in the striatum	[225]
	Paeoniflorin	Cellular model of PD	-	↑viability of neural cells	-	-	↑stability of mitochondrial membranes, ↑brain levels of Paeoniflorin, ↑ATP levels	[233]
	Resveratrol	PD rat model	↓oxidative stress markers, ↑catalase activity and GSH levels in brain	↑stability of neural cells	-	↑cognitive and motor functions	↑activity of ETC complexes	[234]
	Quercetin	PD mouse model	↓lipid oxidation, ↑catalase and SOD activity, ↑GSH levels in hippocampus	-	-	↑memory function, ↓anxious behavior	-	[235]
	Hesperetin	Cellular model of AD	↓activity of cytochrome c as a peroxidase	-	-	-	↑mitochondrial stabilization, ↑ATP levels, ↑activity of ETC complexes	[236, 237]

NLCs: nanostructured lipid carriers, SLN: solid lipid nanoparticles, SPION: superparamagnetic iron oxide nanoparticles, SLN/RVG29/TPP: Solid lipid nanoparticles attached to rabies virus glycoprotein (RVG29) and triphenylphosphonium (TPP) moiety, GSH: glutathione, GSH-PX: glutathione peroxidase, SOD: superoxide dismutase, CAT: catalase, ETC: electron transport chain, Aβ: amyloid β protein, RES: reticuloendothelial system, PD: Parkinson disease, AD: Alzheimer’s disease.

### 5.4. Nanocrystals of Natural Compounds as Antioxidative Agents to Repair Mitochondrial Function in Neurodegeneration

Another way of improving bioavailability and therapeutic efficacy of natural compounds is the formulation as nanocrystals. Nanocrystals are NPs composed of drug particles stabilized by surfactants adsorbed on their surface, which improves the water dissolution and the adsorption of poorly water-soluble compounds by increasing specific surfaces and improving the solubility of saturation [238,239]. Natural compounds such as PUE, QRC, paeoniflorin (PAE), and hesperetin (HESP) are able to repair mitochondrial dysfunction [214,215,223,240,241] and have been formulated as nanocrystals showing a significantly higher recovery of mitochondrial activity along with antioxidative and neuroprotective effects than free compounds in PD and AD models [225,233,234,235,236,237]. Table 3 summarizes the above described nanosystems containing antioxidant and neuroprotective plant chemicals to repair mitochondrial function in ND diseases.

## 6. Restoring Production and Utilization of Mitochondrial Energy in NI&NDDs

Several nanosystems associated with effective agents in restoring mitochondrial energetic production under neurodegenerative conditions have been developed. Ubiquinone or Coenzyme Q10 (CoQ10) is a natural component of mitochondrial ETC and an antioxidant agent with strong evidence supporting its effects on improving mitochondrial energetic function [242]. A nanomicellar water-soluble formulation containing CoQ10, referred to as Ubisol-Q10 restores the impaired ATP production and reduces the elevated levels of mitochondrial ROS in fibroblasts of AD patients [243]. Melatonin, an effective antioxidant and free radical scavenger, associated to PLGA nanocapsules exhibits significantly higher potential than free melatonin to rescue the mitochondrial activity of complex II and antioxidant enzymes in damaged rat brains induced by cerebral ischemia-reperfusion injury [244]. Curcumin encapsulated in SLN shows significant recovery of the activity of mitochondrial complexes and cytochrome levels, significant reduction in mitochondrial swelling, and improvement in neuromotor coordination in a 3-nitropropionic acid-induced HD rat model [245]. Huperzine A (HupA) is an alkaloid from some plants of genus Huperzia used as therapy for memory deficits and acetylcholine-deficit dementia such as AD [246] and to repair mitochondrial function by preserving membrane structural integrity and activity of ETC complexes as well as ATP synthesis in cellular and animal models of AD [247,248]. Nanosystems containing HupA have been described showing enhanced drug delivery compared to free drug or non-targeted NF in mouse brain after intranasal administration [249,250]. In addtion, PEG-PLGA NPs containing HupA show a higher memory recovery than free HupA in AD rats [251]. Therefore, these nanosystems constitute promissory NFs containing proved agents to repair mitochondrial energetic production in neurodegeneration.

Mitochondrial dysfunction results in a reduced ability to generate energy from glucose metabolism in the brain, which along with insulin desensitization observed in the ND brain leads to an increased energetic deficit [252,253,254]. Increased hippocampal oxidative stress, reduced hippocampal mitochondrial OXPHOS coupling efficiency, decreased cortex ATP levels, and cognitive deficits have been found in mice lacking insulin-like growth factor 1 (IGF1) [255]. Consistently, insulin and IGF1 protect mitochondrial structure and upregulate the clearance of defective mitochondria in cellular and mouse models of ALS [256]. Therefore, improving both mitochondrial function and impaired cellular response to insulin might be a key strategy in the treatment of energetic deficit in ND diseases. A nanosystem composed of IGF-1 immobilized on GO-incorporated PLGA electrospun nanofibres used to promote survival, proliferation, and differentiation of neural stem cells [257] might be useful for such purposes. Another alternative would be the use of insulin sensitizers such as Pioglitazone (PIO), which is a therapeutic option for managing type 2 diabetes mellitus [258]. PIO reduces oxidative damage and restores mitochondrial energy production and ETC complex activity in the hippocampus and cortex of AD mouse models [259,260]. Cerebral blood flow and glucose uptake are significantly improved in AD mice after long-term oral treatment with PIO [261]. Interestingly, compared to free PIO, PLGA NPs loaded with PIO induce a significantly higher mitochondrial respiratory activity leading to higher ATP production in a cellular AD model [262]. Similarly, biguanide Metformin (MET), another insulin sensitizer, reduces mHTT protein levels, ameliorates cognitive and motor alterations observed in a HD (Hdh150) mouse model [263] as well as attenuated memory deficit and neuron loss, and enhanced neurogenesis in the hippocampus of transgenic AD mice [264]. At a mitochondrial level, MET positively impacts mitochondrial dynamics by restoring blocked mitophagy, promoting mitochondrial biogenesis, and improving respiratory activity through activation of AMPK, SIRT1, and PARKIN in cellular models of mitochondrial dysfunction [265,266,267]. Remarkably, synthetic mitochondria-targeted metformin derivatives conjugated to the TPP moiety [268,269] produce a neuroprotective effect and improve motor deficits restoring dopamine levels in the MitoPark transgenic mouse model of PD [270].

In addition to insulin sensitizers, incretin peptides such as glucagon-like peptide (GLP) and glucose-dependent insulinotropic peptide (GIP) promote insulin secretion and re-activation of energy utilization [271] and they constitute promissory therapeutic tools on mitochondrial dysfunction and efficient brain glucose utilization. Diverse GLP and GIP receptor agonists have shown direct effects on mitochondrial function by restoring the energetic imbalance associated with a reduction of brain inflammation and cognitive decline under ND conditions [272,273,274,275,276,277,278,279]. Importantly, some of these GLP agonists have also been effective in attenuating neuroinflammation in a mouse model of MS [279,280]. Interestingly, several nanosystems containing incretin analogs have been developed [281,282,283,284,285,286,287] and they represent promising therapeutic strategies to be evaluated in different models of NI&NDD. Table 4 summarizes the described nanosystems containing regulators of mitochondrial energetic function in ND models.

## 7. Reducing the Impact of Proteinopathies on Mitochondria

As discussed above, the accumulation of protein aggregates impacts mitochondrial structure and function. Hence, different nanosystems have been developed to prevent mitochondrial dysfunction by reducing formation and accumulation of toxic peptide aggregates under ND conditions (Table 5).

### 7.1. Interfering with Protein Aggregation

The most direct and simple strategy to overcome mitochondrial and cell damage derived from toxic protein accumulation is limiting aggregation by using anti-aggregative agents. Diverse organic and inorganic nanomaterials and nanosystems have been described as effective at interfering with peptide fibrillogenesis either by direct interaction with the peptide or by inducing conformational changes of monomers that prevent further aggregation. Due to their large accessible pore size and easy release of incorporated compounds from pores, porous AuNPs and porous silica NPs are effective inhibitors of mHTT aggregation in a SH-SY5Y cell model of HD [289]. Carbon-based nanomaterials are effective anti-aggregative agents acting on toxic peptides associated with ND pathologies. For instance, graphene quantum dots (GQD) and GO show effective inhibition of Aβ peptide accumulation in a cell free system, and rescue cell viability from Aβ-mediated cellular toxicity in PC12 cells [290,291]. Moreover, GQD and GO disrupt the α-syn aggregation rate and interfere with the progression of α-syn fibrillation [293]. In vivo experiments show that GQD treatment reduces the aggregation of α-syn and the loss of dopaminergic neurons and attenuates motor deficits in mice after stereotaxical injection of α-syn fibrils [294]. Importantly, the anti-aggregative effect of GDQ was associated with the alleviation of mitochondrial damage (shrinkage, decreased oxygen consumption rate, and elevated ROS levels) in mouse cortical neurons treated with α-syn preformed fibrils [294].

The dissolution or inhibition of these protein aggregates results in a large amount of free monomers, which are still toxic molecules able to further aggregate. Hence, further degradation of toxic proteins is needed. GO treatment prevents the accumulation Aβ aggregates by inhibiting β-cleavage of amyloid precursor protein (APP) and also activates its degradation by promoting endosomal Aβ delivery to lysosomes in a cellular model overexpressing APP (HEK293T-APP and SHSY5Y-APP cells) [296]. A nanosystem composed of MET loaded in polydopamine promotes the proteasomal degradation of phosphorylated α-syn in a cellular PD model [297].

The C-terminal region of TDP-43 includes a Gln/Asn-rich segment essential for its aggregation [310,311]. Polyglutamine binding peptide 1 (QBP1) recognizes this segment and inhibits TDP-43 aggregation [312]. Although the impact of its anti-aggregative effect on TDP-43 on mitochondrial biology has not been assessed, QBP1 shows broad activityto block the aggregation of diverse polypeptides affecting mitochondrial function such as mHTT and α-syn [313,314]. A nanosystem made of PLGA NPs coated with polysorbate 80 and containing QBP1 showed dose dependent inhibition of mHTT aggregation in cellular models of HD and improved motor performance in a *Drosophila* model of HD [295]. Similarly, a nanosystem of PEG-block-polycaprolactone (PEG-*b*-PCL) NPs loaded with QBP1 showed effective suppression of cell death in a cellular model of HD. The same concentration of free QBP1 peptide were therapeutically ineffective [315].

The phosphorylation of peptides associated with ND proteinopathies is a key process for their toxic aggregation, accumulation, and further cognitive deficits [93,316,317,318], which has been addressed by various studies. A metallic nanosystem of AuNPs reduces Tau phosphorylation and improves mitochondrial antioxidant balance and ETC complex activity along with the recovery of cognitive impairment in a rat AD model induced by intracerebral administration of okadaic acid [288]. Fingolimod (FTY720), a drug used for the treatment of MS [319] induces activation of protein phosphatase-2, α-syn dephosphorylation, and attenuation of PD progression [320]. Notably, a NF of chitosan containing FTY720 is more effective than free FTY20 in attenuating mitochondrial alterations associated with inhibition of α-Syn phosphorylation in an in vitro rotenone-induced PD model [298].

### 7.2. Nanosystems Containing Natural Compounds as Mitochondrial Agents for the Treatment of Proteinopathies in Neurodegeneration

An important factor contributing to the aggregation and accumulation of toxic peptides is the oxidative environment [321,322,323]. Agents with antioxidant properties involved in mitochondrial function also provide neuroprotection by the promoting disaggregation of toxic proteins [306,307,324,325]. A wide variety of plant extracts and phytochemicals with antioxidant properties inhibiting aggregation of peptide oligomers have been reviewed elsewhere [326,327]. In order to improve their bioavailability and therapeutic effect, recent nanosystems containing natural compounds have been developed and evaluated for their ability to disaggregate toxic peptides associated with mitochondrial dysfunction in ND models. EGCG, a natural polyphenol component of green tea, promotes mitochondrial biogenesis by activating the PGC1ɑ signaling pathway [328,329]. A NF made of poly lactic acid (PLA) fused to PEG containing EGCG administered by the oral route results in significant restoration of cognitive deficits and reduction of the accumulation of Aβ aggregates in the hippocampus of an AD rat model induced by aluminum chloride [300]. Similarly, EGCG conjugated to the surface of micellar structures based on superparamagnetic iron oxide NPs (spions) shows a neuroprotective effect and a considerable inhibition of α-syn aggregation in a mouse model of PD [301]. Likewise, EGCG loaded on PEGylated PLGA NPs reduces the cortical levels of soluble and insoluble Aβ to a higher extent than free EGCG in a transgenic mouse model of AD. These biochemical changes are accompanied by the restoration of impaired learning and memory [302]. Chlorogenic acid (CGA), a natural phenolic acid, shows antioxidative and neuroprotective effects associated with the recovery of ETC complex activity and the increase of mitochondrial antioxidant defense in the brain of PD mice [324]. A nanosystem of selenium NPs containing CGA was more effective than free CGA in reducing ROS levels, preventing amyloid aggregation, and reducing the death of Aβ treated PC12 cells [304]. Similar results were reported with a nanosystem of selenium NPs and QRC in PC12 cells treated with H_2_O_2_ [303]. In these studies, the antioxidant effect is provided by both the plant polyphenol (e.g., CGA or QRC) and the selenium as an agent that promotes cellular redox regulation and prevents the neurotoxicity of Aβ [330,331].

Diverse curcumin NFs showed superior effects against α-syn aggregation, fibrillation, and cellular toxicity as compared to free curcumin. For instance, a liposomal nanocarrier for curcumin coated with polysorbate 80 ameliorates motor deficits and improves dopamine expression by promoting α-syn clearance in a PD mouse model induced by MPTP treatment [305]. A carbonyl analogue of curcumin self-assembled in a nanosystem with PEG, but not the free molecule, afforded neuroprotection in cellular models of PD [306]. More importantly, intranasal delivery of the curcumin analogue ameliorates behavioral deficits and promotes clearance of monomers, oligomers, and aggregates of α-syn in the midbrain of an MPTP mouse model of PD [306].

Baicalein (5,6,7-trihydroxyflavone), a bioactive flavone of *Scutellaria baicalensis Georgi*, has been found to have neuroprotective effects in PD models in vivo and in vitro by suppressing the accumulation of α-syn aggregates [332,333]. In parallel, baicalein has also been shown to enhance mitochondrial biogenesis in a rotenone-induced PD rat model [334]. More importantly, baicalein associated to nanoliposomes made of DPPC (1,2-dipalmitoyl-*sn*-glycero-3-phosphocholine), PEG and cholesterol (NLP-Ba) prevents α-syn fibrillation and also depolymerizes mature fibrils more effectively than free baicalein in the substantia nigra pars compacta of mouse brains treated with rotenone as model of PD [307].

Berberine, an isoquinoline alkaloid, has shown neuroprotective effects by counteracting mitochondrial dysfunction in in vitro and in vivo models of ND diseases [335,336,337,338]. Interestingly, a higher reduction of the elevated levels of Aβ and Tau and improvement of learning and memory deficits were obtained with a NF made of chitosan NPs containing berberine, but not with the free drug, in scopolamine-treated AD rats [308]. Acteoside (ACT) (verbascoside), a phenolic phenylpropanoid glycoside, inhibits aggregation of Aβ in a cell-free system [339] and improves mitochondrial morphology and function in Aβ-exposed neuronal and astrocyte cells [340,341]. A NF made of chitosan and PEG-PLA NPs containing ACT and conjugated with nerve growth factor as a targeting moiety to neurons reduces α-syn aggregates in PC12 cells and in the substantia nigra of MPTP-treated PD mice to a greater extent than the free drug or blank nanosystems. The amelioration of α-syn levels is accompanied by the prevention of dopaminergic neuron death and improved motor deficits of PD mice [309].

## 8. Therapeutic Nanomaterials for Mitochondrial Dysfunction in NI&NDDs

Novel organic and inorganic materials with modulatory effects on mitochondrial dysfunction and neurodegeneration or used for the synthesis of more efficient nanosystems for the delivery of mitochondrial drugs have been described and evaluated in cellular and animal models of ND disorders (Table 6).

### 8.1. Photothermal Nanomaterials

Nanomaterials with photothermal effect (PTE) convert near-infrared (NIR) light into heat, inducing a local hyperthermia used for nanocarrier disintegration and release of active drug and/or disaggregation of protein aggregates, preventing their accumulation and interference with mitochondria [342,361,362]. Au nanorods (AuNRs) coated with mesoporous silica and associated with QRC activated with NIR light restored intracellular ATP levels and MMP in an in vitro PD model and restored dopaminergic neurons and motor deficits in a MPTP-induced mouse model of PD [342]. Black phosphorous (BP) is a nanomaterial of planar layers of phosphorous bonded to each other by van der Waals forces [363]. BP structures offer a larger specific surface area than graphene nanosystems to obtain a superior loading capacity for drug delivery [363,364] and possess effective PTE, which can increase permeability through biological barriers allowing controlled drug delivery upon NIR irradiation [343]. BP nanosheets exert antioxidant effects by capturing Cu^+2^ and suppressing Cu^+2^-catalyzed redox reactions, thereby preventing mitochondrial damage in neuronal SH-SY5Y cells [343]. The in vivo administration of brain-targeted lactoferrin-BP nanosheets loaded with PAE localized to mitochondria improves antioxidant balance and restores DA levels in response to NIR irradiation in the striatum of a mouse PD model. These biochemical changes are accompanied by significant improvements in the mobility deficits of PD mice [197].

### 8.2. Nanozymes: Nanomaterials with Enzymatic Activity

Nanozymes are organic and inorganic nanomaterials with enzymatic-like activities such as peroxidase (Px), glutathione peroxidase (GSH-Px), catalase (CAT), antioxidant glutathione (GSH), and SOD [350,365]. Unlike biological enzymes, nanozymes show higher stability in a wide range of pH and temperatures, and the formulations can be produced at low cost and at large scale [365]. Diverse nanozyme materials have emerged as novel therapeutic tools for ND diseases, mimicking the function of natural antioxidant enzymes and counteracting mitochondrial dysfunction. Nanozymes of pure metallic materials have been reported as effective agents in the degradation and prevention of peptide aggregates potentially toxic for mitochondrial function. For instance, polyoxometalates (POMs) are negatively charged metal-oxygen structures associated with transition metal ions. POMs are effective in the degradation and prevention of toxic effects of Aβ displaying both protease-like activity for Aβ disaggregation and SOD-like activity to scavenge ROS produced by Aβ reactivity [344]. POM nanostructures associated with Niobium act as potent inhibitors of S100A9 assembly [345], which is a protein present in neuron cells and involved in the aggregation of Aβ and α-syn [366,367] and related to cognitive deficits in an AD animal model [368]. A bimetallic nanosystem composed of a nanozyme core of platinum-copper (PtCu) and coated with polyvinyl pyrrolidone significantly inhibits α-syn aggregation and spread in the brain of mice after intrastriatal injection of α-syn fibrils. The neuroprotective effect of the PtCu nanosystem is associated with the strong antioxidant properties mediated by its Px, CAT and SOD-like activity [346].

In another study, metallic NPs of palladium hydride (PdH) with catalytic hydrogen production and the ability to scavenge •OH radicals were able to reduce oxidative stress and the accumulation of Aβ and promote recovery of mitochondrial dysfunction by enhancing the expression of ETC cytochrome C oxidase subunit IV and the fusion protein Mfn2 as well as decreasing the levels of fission protein DRP1 in the brain of transgenic AD mice. These biochemical changes correlate with the recovery of cognitive impairment in AD [347]. A metallic suspension of nanocrystals of Au with catalytic activity on NADH oxidation, referred to as CNM-Au8, and administered orally, promotes remyelinating activity in two demyelinating mouse models of MS induced by cuprizone or lysolecithin treatment. The remyelinating effect provided by CNM-Au8 was accompanied by an increased expression of myelin-synthesis related genes in oligodendrocyte precursor cells as well as elevated levels of total intracellular ATP and redox coenzyme nicotine adenine dinucleotide (NAD^+^) in primary co-cultures of neural and glial cells obtained from the mesencephalon of rat embryos [348]. An acute deficiency of NAD^+^ was found in serum samples of MS patients, which is related to a more severe clinical course of the disease [369]. Currently, two phase 2 clinical trials are assessing the energetic improvement, efficacy, safety, pharmacodynamics, and pharmacokinetics of CNM-Au8 therapy in MS (NCT03993171) and ALS (NCT04098406) [370] patients. A phase 1 clinical trial showed safe and well-tolerated outcomes in healthy volunteers receiving CNM-Au8 treatment (NCT02755870).

Metal oxide compounds displaying SOD, CAT, and hydroxyl radical scavenging activities [371] constitute another category of metallonanozymes used to repair mitochondrial function and neurodegeneration in different models of ND disorders [349,350]. Treatment with Mn_3_O_4_ nanozymes counteracts mitochondrial dysfunction by reducing mitochondrial oxidative status, swelling, and mPTP opening as well as restoring MMP, and increasing the activity of ETC complexes and ATP levels in the brain of HD mice [349]. Additionally, Mn_3_O_4_ nanozymes recover motor, cognitive and behavioral impairment [349]. Likewise, cerium oxide (CeO_2_) NPs displaying SOD, CAT, and Px-like activities depending on the dominant electronic state between Ce^+4^ and Ce^+3^ [372] have been assessed as modulators of oxidative status and mitochondrial dysfunction in cellular and animal models of AD [373], PD [163] and MS [374]. CeO_2_ NPs were able to compensate SOD deficiency and prolong lifespan in an ALS mouse model expressing a mutant and inactive SOD protein [351]. CeO_2_ NPs inhibit cytoplasmic α-syn accumulation and counteract mitochondrial fragmentation and ROS production in a yeast model of PD expressing human α-syn [352,375]. In primary cortical neurons from rat, CeO_2_ NPs were localized at OMM and ameliorated mitochondrial fragmentation and neuronal cell death by quenching O_2_·^−^ and limiting peroxynitrite formation [151]. In an interesting dual system, SOD-like activity of CeO_2_ was combined with the proteolytic activity of POM to produce a synergic nanozyme (CeONP-POMs) that was able to induce effective attenuation of oxidative damage along with the degradation of Aβ aggregates in vitro, inhibition of Aβ-induced toxicity in PC12 cells, and suppression of Aβ-induced BV2 microglial cell activation. Importantly, in vivo experiments showed that CeONP-POMs crossed the BBB and did not present toxicity [353]. Nanorods of cerium vanadate (CeVO_4_) with SOD-like activity prevent oxidative damage to mitochondria and restore mitochondrial integrity in SH-SY5Y cells deficient in SOD1 and SOD2 activity [354]. A mitochondria-targeted nanozyme formulation composed of MoS_2_ quantum dots with SOD and CAT activities and associated with TPP (MoS_2_-TPP) reduces ROS levels, prevents structural alterations and the degradation of mitochondria, and promotes the transition from an inflammatory M1 phenotype to an anti-inflammatory M2 phenotype in Aβ-treated BV-2 microglial cells. Furthermore, in vivo experiments showed that MoS_2_-TPP reaches the brain, increases the neuroprotective protein NEuN, reduces oxidative stress, ameliorates Aβ aggregation in the hippocampus, and promotes the microglial transition from the proinflammatory M1 state to the tissue-repairing M2 phenotype in transgenic (APP/PS1) AD mice [355].

A metal oxide nanozyme made of Cu(II) ions coordinated to phenylalanine as a basal structure (Cu_x_O NPs) and displaying CAT, GSHPx, and SOD activities promotes cytoprotection against oxidative-stress-mediated neurotoxicity in SHSY-5Y cells and reduces oxidative stress, restores dopamine levels in brain tissue, and improves memory function in a MPTP-induced PD mouse model [356].

Other nanomaterials derived from carbon structures such as graphene derivatives also show nanozyme properties. GOQD displays antioxidant function through CAT-like activity reducing ROS levels, promoting mitochondrial protection, preventing neuronal death, and increasing motor activity in a PD zebrafish model [357]. Carboxyfullerene (C60), a water soluble derivative of fullerene (icosahedrons structures with 60 carbon atoms and a 0.7 nm diameter) that behaves as a free-radical scavenger [376] associates with mitochondria [359,377] and suppresses the neuroinflammatory response in lipopolysaccharide (LPS)-stimulated murine BV-2 microglial cells, thereby inhibiting ROS generation and stabilizing mitochondrial membranes [358]. Due to its SOD-like activity, C60 treatment increases the lifespan of mice deficient in mitochondrial manganese SOD [359] and reduces superoxide levels in isolated mitochondria from brain tissue of aged mice [378]. In a transgenic mouse model of ALS expressing a mutant form of SOD, C60 treatment results in delayed symptom onset and improves survival [379]. In addition, C60 was effective in reducing striatal injury, restoring dopamine levels, and improving motor function in a PD primate model [380]. More recently, it has been reported that C60 treatment reverts mitochondrial dysfunction by restoring the activity of ETC complexes, ROS scavenging and GSH levels in the brain and skeletal muscle mitochondria of a HD rat model induced by 3-NPA treatment [360].

## 9. Conclusions and Future Perspectives

Mitochondria play a direct and indirect role in NI&NDDS. Their structural and functional integrity are involved in the stability and viability of neural tissue. Thus, mitochondria-targeted therapy is a potential strategy to treat NI&NDDs diseases. Developing and evaluating new mitochondrial treatments based on nanotechnology would improve the costs and therapeutic efficacy by controlled, sustained, and specific delivery of reduced amounts of active drugs.

The energy impairment resulting from mitochondria alteration in NI&NDDs also affects energy metabolism in cells and neural tissues containing these altered organelles. An effective therapy should consider not only the local effects on mitochondrial integrity but also the correction of impaired glucose metabolism that limits the production of more energy to support the demands of the neural tissue. There is potential for research on glucose-lowering therapies used in diabetes, which may be useful to treat NI&NDDs such as nanosystems of insulin sensitizers and incretin peptides [271].

Remarkably, there is also a wide diversity of plant chemicals and nanomaterials to explore as efficient therapeutic mitochondrial agents with reduced costs and side effects. The ample flexibility of nanosystems and new nanomaterials as well as tissue and cellular targeting allows the design of superior nanocarriers and improved physicochemical characteristics for the persistent and efficient delivery of therapeutic compounds.

## Figures and Tables

**Table 1:** Protein aggregates related to mitochondrial dysfunction in models of neurodegenerative diseases.

Protein Aggregates	Disease Model	Effect on Mitochondria	Refs
α-synuclein	PD	↑mitochondrial ROS levels, ↓ ETC activity, ↓stability of mitochondrial membranes, ↑mPTP opening, ↓mitochondria-ER contacts, ↑DRP1 and ↓mitochondrial SIRT3 levels (a protective molecule of mitochondrial integrity and energetic function [84])	[72,73,74,83,100]
Amyloid b	AD	↑mitochondrial ROS levels, ↑mitochondrial fission (↓Mfn1, ↑DRP1 levels and ↑O-GlcNAcylation of DRP1)	[68,69,91, 92,94]
Tau	AD	↑microtubule dissociation, ↑mitochondrial ROS levels, ↓ATP production, ↑mitochondrial fission and ↓mitophagy (interaction with DRP1 and PARKIN proteins)	[70,71,93]
Transactive response DNA-binding protein of 43 kDa (TDP-43)	AD, ALS	↑mitochondrial ROS levels, ↓stability of mitochondrial structure, ↑mPTP opening	[61,75,95,96,97]
Huntingtin	HD	↑mitochondrial ROS levels, ↓stability of mitochondrial structure, ↑mPTP opening, ↑mitochondrial fission by activation of DRP1, ↓mitophagy, ↑disruption Ca^+2^ flux between ER and mitochondria	[49,79, 80,101]
Superoxide dismutase	ALS	↓mitophagy (by arresting optineurin protein), ↓stability of mitochondria structure, ↓flux of protein from and to the mitochondria	[102,103,104,105]

ROS: reactive oxygen species, SIRT3: sirtuin 3, DRP1: dynamin related protein, α-syn: α-synuclein, ETC: electron transport chain, ER: endoplasmic reticulum, mPTP: mitochondrial permeability transition pore, Mfn1: mitofusin 1, PARKIN: PARKIN RBR E3 ubiquitin-protein ligase, PD: Parkinson’s disease, AD: Alzheimer’s disease, ALS: amyotrophic lateral sclerosis, HD: Huntington’s disease.

**Table 2:** Nanosystems containing antioxidant agents to repair mitochondrial function in models of neurodegenerative diseases.

Nanosystem	Drug	Disease Model	Effect on Mitochondria and Neurodegeneration					Refs
			ROS Levels	Selective Localization of Drug	Cell Viability	Inflammation	Other Effects	
DQAsome/ P68	NAC/ DFO	Cellular model of PD	↓ (cellular and mitochondrial)	-	↓neuronal death	-	↑cellular antioxidant activity, ↓total non-ferritin-bound iron	[153]
CeO_2_	Cerium	Cellular model of AD	↓ (cellular and mitochondrial)	↑ (mitochondrial)	↓neuronal death	-	↓DRP1 activation, ↓mitochondrial fission, ↓tyrosine nitration	[151]
CeO_2_-TPP	Cerium	Cellular model of AD	↓ (cellular and mitochondrial)	↑ (mitochondrial)	↓neuronal death	↓glial cell activation, ↓lipid peroxidation	↑mitochondrial stabilization	[162]
CeO_2_-TPP	Cerium	PD mouse model	↓ (cellular and mitochondrial)	↑ (mitochondrial)	-	↓glial cell activation, ↓lipid peroxidation	↑mitochondrial stabilization, ↑brain tyrosine hydroxylase levels (enzyme that converts L-tyrosine into levodopa, a precursor of dopamine)	[163]
PLGA-bPEG-TPP	Curcumin	Cellular model of AD	-	↑ (mitochondrial)	↑neuronal viability	-	↑endosomal and lysosomal escape	[168]
HSA/RBC/ t807/-TPP	Curcumin	Cellular and mouse model of AD	↓ (cellular and mitochondrial)	↑ (neuronal and mitochondrial)	↑neuronal survival in hippocampus	↓glial cell activation	↑long-term circulation, ↑learning and memory function	[169]
Nanomicelles	TEMPO (4-amino-2,2,6,6-tetramethylpiperidinyloxy)	AD mouse model	↓ (cellular)	-	-	↓lipid peroxidation, ↓brain Aβ accumulation	↓oxidative stress, ↑learning and memory function	[172]
		Cellular model of PD	↓ (cellular and mitochondrial)	-	↑neuronal viability at pH 6,5	-	↑mitochondrial mass, ↑GSH content, ↑mitochondrial stabilization, ↑ATP levels	[173]
Nanomicelles	Carnosine/ lipoic acid	PD mouse model	-	-	-	↓lipid peroxidation	↑antioxidant activity in the brain tissue, ↑levels of dopamine and serotonin	[176]
Gold nanoparticles	Lipoic acid	Cellular model of PD	↓ (cellular)	-	↑neuronal viability	↓lipid peroxidation	↑ATP levels and mitochondrial respiratory activity, ↑cell membrane and microtubule stabilization	[177]

DQAsome: dequalinium nanocarrier, P68: Pluronic F68, NAC/DFO: N-acetyl cysteine/deferoxamine, CeO_2_: cerium oxide, CeO_2_-TPP: cerium oxide attached to triphenylphosphonium, PLGA-bPEG-TPP: poly (L-lactic-co-glycolic acid) and poly (ethylene glycol) fused to triphenylphosphonium, HSA/RBC/t807/-TPP: human serum albumin NPs (HAS) fused to triphenylphosphonium and coated with a shell of red blood cell membrane (RBC) and 7-(6-nitropyridin-3-yl)-5H-pyrido[4,3-b] indole moiety (T807), ROS: reactive oxygen species, DRP1: dynamin related protein, Aβ: amyloid β protein, α-Syn: α-synuclein protein, 6-OHDA: 6-hydroxydopamine, PD: Parkinson disease, AD: Alzheimer disease.

**Table 4:** Nanosystems containing regulators of mitochondrial energetic function in models of neurodegenerative diseases.

Nanosystem	Drug	Disease Model	Effect on Mitochondria and Neurodegeneration			Refs
			Oxidative Stress	Energetic Function	Other Effects	
Nanomicellar CoQ_10_	Ubiquinone	Fibroblasts from AD patients	↓cellular and mitochondrial ROS	↑ATP levels	delays the onset of premature senescence	[243]
PLGA NPs	Melatonin	Ischemia–reperfusion injury	↓mitochondrial oxidative stress, restoration of SOD, CAT and GSH-Px activity	↑activity of ETC complexes	↑stability of mitochondrial membranes, ↑stability and survival of pyramidal neurons	[244]
SLN	Curcumin	HD rat model	↓cellular ROS, lipid peroxidation and protein oxidation. ↑mitochondrial GSH and SOD activity	↑activity of ETC complexes and cytochrome levels	↓mitochondrial swelling, ↑neuromotor coordination	[245]
PLGA-PEG NPs	Huperzine A	AD rat model	-	-	↑memory and cognitive recovery, ↑bioavailability	[251]
PLGA NPs	Pioglitazone	Cellular model of AD	-	↑mitochondrial respiratory activity, ↑ATP levels	↑brain bioavailability. Modulation of locomotor activity and brain energetic metabolism	[262]

PLGA: poly (L-lactic-co-glycolic acid), PLGA-PEG: poly (L-lactic-co-glycolic acid) fused to poly (ethylene glycol), SLN: solid lipid nanoparticle, ROS: reactive oxygen species, GSH-PX, glutathione peroxidase, SOD: superoxide dismutase, CAT: catalase, ETC: electron transport chain.

**Table 5:** Nanosystems to reduce the biological impact of proteinopathies in models of neurodegenerative diseases.

Role of Nanosystem	Nanosystem Composition	Disease Model	Effect on Proteinopathies, Mitochondria and Neurodegeneration				Refs
			Oxidative Stress	Protein Aggregates	Cell Viability	Other Effects	
Dissolution or inhibition of protein aggregation	Inorganic, Gold NPs	AD rat model	↓oxidative markers, ↑antioxidant status (SOD and catalase activity and GSH levels)	↓phosphorylation and Tau levels	-	↓inflammatory markers, ↑ETC complex activity, ↑ATP levels, ↑cognitive functions	[288]
		Cellular model of HD	-	↓mHTT aggregation	-	↓amyloid aggregation of insulin	[289]
	Inorganic, porous silica NPs	Cellular model of HD	-	↓aggregation and accumulation of mHTT	-	↓amount of aggregated amyloid of insulin and ↓insulin amyloid fibrillation	[289]
	Organic, graphene nanosystem (GQD, GO)	Cellular AD model	-	↓Aβ aggregation,	↑cell viability from Aβ-mediated cellular toxicity	↑preservation of mitochondrial function	[290,291,292]
		Cellular and mouse models of PD	↓cellular and mitochondrial ROS induced by α-syn in neurons	↓α-syn aggregation, ↑dissociation of α-syn fibrils	↓loss of dopaminergic neurons induced by α-syn	↓mitochondrial damage in α-syn-treated primary cortical neurons, ↑preservation of mitochondrial function, ↓glial activation, ↓motor deficits	[293, 294]
	Organic, PLGA/polysorbate80- QBP1	Cellular and fly models of HD	-	↓mHTT aggregation	-	↑motor performance in *Drosophila* model of HD	[295]
Degradation of toxic proteins	Organic, GO nanosheet	Cellular model of AD	-	↓accumulation of hippocampal Aβ aggregates, ↑Aβ delivery to lysosomes for degradation	-	↓β-cleavage of amyloid precursor protein (APP) by BACE1 activity	[296]
	Organic, Polydopamine NPs/ metformin	Cellular model of PD	↓cellular ROS levels	↑proteasomal degradation of phosphorylated α-syn	↓neuron loss induced by rotenone	↓inflammatory markers, ↑stabilization of mitochondrial membranes	[297]
Inhibition of protein phosphorylation	Chitosan NPs/ Fingolimod	Cellular model of PD	↓cellular ROS levels	↓phosphorylation and accumulation of α-syn	↑cell viability of rotenone-treated neurons	↑stability of mitochondrial membranes	[298]
	PLGA NPs/PHA-767491	Cellular model of ALS	↓cellular and mitochondrial ROS levels	↓phosphorylation and accumulation of TDP-43	↓loss of dopaminergic neurons	↑permeability through BBB in a cellular model	[299]
Therapeutics against protein aggregation based on natural compounds	PLA-PEG NPs/EGCG	AD rat model	↓cellular ROS levels	↓accumulation of Aβ aggregates in the hippocampus	-	↑locomotor and cognitive abilities	[300]
	Micellar SPIONS/ EGCG	PD mouse model	EGCG release upon oxidative conditions	↓α-syn aggregation	↑dopaminergic neurons	↑permeation through a BBB model, ↑motor and cognitive abilities	[301]
	PLGA-PEG NPs/EGCG	AD mouse model	-	↓levels of soluble and insoluble Aβ peptide	-	↑permeation through a BBB model, ↓glial activation in cortex and hippocampus, ↑learning and memory abilities	[302]
	SeNPs/CGA or QRC	Cellular model of AD	↓ROS levels	↓Aβ aggregation	↓cell death induced by Aβ	↑mitochondrial stability, ↓release of mitochondrial lactate dehydrogenase	[303, 304]
	Liposomal-polysorbate80/ curcumin	PD mouse model	-	↓α-syn accumulation in brain tissue	-	↑curcumin brain accumulation and circulation lifetime, ↑dopamine levels, ↑motor function	[305]
	PEG- carbonyl curcumin	Cellular and mouse model of PD	-	↑α-syn lysosomal degradation in vitro, ↓α-syn accumulation in brain tissue	↓cell death of dopaminergic neurons	↑memory and motor function	[306]
	PEG- cholesterol-Nanoliposomes/ baicalein	Cellular and mouse model of PD	↓ROS levels in cellular model of PD	↓α-syn fibrillation, ↑disaggregation of α-syn fibrils	↑neurite growth, ↑dopamine levels and stability of dopaminergic neurons	↑motor function in PD mouse model	[307]
	Chitosan NPs/berberine	AD rat model	↓oxidative stress in brain tissue	↓levels of Aβ and Tau	-	↑learning and memory abilities	[308]
	Chitosan-PLA-PEG-NGF NPs/ Acteoside	PD mouse model	-	↓α-syn aggregation in cells and brain of PD mice	↑stability of dopaminergic neurons	↑dopamine levels of dopaminergic neurons, ↑motor function	[309]

GQD: graphene quantum dots, GO: graphene oxide, PLGA/polysorbate80- QBP1: poly (l-lactic-*co*-glycolic acid) coated with polysorbate 80 and loaded with polyglutamine binding peptide 1 (QBP1), PLGA NPs/PHA-767491: poly (l-lactic-*co*-glycolic acid) nanoparticles loaded with (1,5,6,7-tetrahydro-2-(4-pyridinyl)-4H-pyrrolo[3,2-c]pyridin-4-one hydrochloride) (PHA-767491), PLA-PEG NPs/EGCG: poly lactic acid fused to poly (ethylene glycol)(PEG) and loaded with epigallocatechin-3-gallate, PLGA-PEG NPs/EGCG: poly ((l-lactic-co-glycolic acid) fused to poly (ethylene glycol)(PEG) and loaded with epigallocatechin-3-gallate, Micellar SPIONS/EGCG: micelles of superparamagnetic iron oxide nanoparticles associated to epigallocatechin-3-gallate, SeNPs/CGA or QRC: selenium nanoparticles associated to chlorogenic acid or quercetin, Chitosan-PLA-PEG-NGF NPs/Acteoside: Chitosan and poly lactic acid fused to poly (ethylene glycol) nanoparticles containing Acteoside and conjugated to nerve growth factor (NGF), ETC: electron transport chain, ROS: reactive oxygen species, α-syn: α-synuclein, Aβ: amyloid β, mHTT: mutant huntingtin protein, TDP-43: transactive response DNA-binding protein of 43 kDa, BBB: blood-brain barrier, PD: Parkinson’s disease, AD: Alzheimer’s disease, ALS: amyotrophic lateral sclerosis, HD: Huntington’s disease.

**Table 6:** Therapeutic nanomaterials for mitochondrial dysfunction in models of neurodegenerative diseases.

Nanomaterial	Nanosystem Composition	Disease Model	Effect on Mitochondria and Neurodegeneration					Refs
			Oxidative Stress	Mitochondrial Function	Cell Viability	Protein Aggregates	Other Effects	
Photothermal	Gold nanorods coated by mesoporous silica and associated to quercetin	Cellular and mouse models of PD	-	↑ATP levels, ↑stability of mitochondrial membranes	↑preservation of dopaminergic neurons	-	↑permeability through a BBB model, ↑brain accumulation, ↑striatal dopamine levels, ↑motor performance	[342]
	Black Phosphorous	Cellular and mouse models of Cu toxicity	↓cellular oxidative stress and damage	↑stability of mitochondrial membranes	↑neural cell viability under oxidative stress conditions	-	↑permeability through a BBB model, ↑brain accumulation	[343]
		Cellular and mouse models of PD	↑antioxidant status (↓lipid oxidation, ↑SOD activity and GSH levels)	↑mitochondrial localization	↑preservation of dopaminergic neurons	-	↑permeability through a BBB model, ↑brain accumulation, ↑dopamine levels, ↑motor function	[197]
Metallic nanozymes	Polyoxometalates	Cellular and cell-free models of AD	↓cell ROS levels induced by Aβ, ↑ability to scavenge oxidant Cu ions, ↑SOD-like activity	-	↑cell viability under Aβ toxicity	↑degradation of Aβ fibrils and aggregates, ↓assembly of amyloid S100A9 protein	↑permeability through a BBB model	[344, 345]
	Platinum-copper/ polyvinyl pyrrolidone	Cellular and mouse models of PD	↓cell ROS levels, ↑activity of antioxidant-like enzymes	-	↑cellular neuroprotection by expression of NeuN protein	↓α-syn aggregation and spreading	-	[346]
	Palladium hydride	AD mouse model	↓cellular ROS levels	↑expression of ETC complex IV, ↑mitochondrial respiratory activity, ↓levels of fission protein DRP1, ↑expression of fusion protein Mfn2	↓apoptotic pathway	↓Aβ aggregation	↑cognitive function	[347]
	Nanocrystals of gold (CNM-Au8)	Cellular and mouse models of MS	-	↑ATP and NAD^+^ levels in oligodendrocyte precursor cells	↑differentiation of oligodendrocytes from precursor cells	-	↑remyelinating activity, ↑expression of myelin synthesis-related genes, ↑motor functions	[348]
Metal oxide nanozyme	Flower-like Mn_3_O_4_	Cellular model of PD and mouse model of HD	↓mitochondrial ROS and lipid oxidation, ↑GSH peroxidase-like activity and brain antioxidant activity	↑brain activity of ETC complexes and ATP levels, ↓mitochondrial swelling and mPTP opening, ↑stability of mitochondrial membranes	↑preservation of brain cell structure, ↓focal degeneration of neural cells	-	↑motor and cognitive functions, ↓anxious behavior	[349, 350]
	Cerium oxide NPs	Mouse model of ALS	↑catalase-like activity in cell-free system, ↓oxidative stress in brain tissue	-	↑cell viability under oxidative conditions	-	↑muscle strength, motor function and mouse lifespan	[351]
		Yeast model of PD	↓ROS levels	↓mitochondrial fission	↑cell viability under α-syn damage	↓α-syn aggregation and accumulation	-	[352]
		Cellular model of AD	↓oxidative stress and formation of peroxynitrite	↓mitochondrial fission, ↓DRP1 activation	↓neuronal cell death under oxidative stress and Aβ treatment	-	↑localization in mitochondria, ↑protein tyrosine nitration	[151]
	Cerium oxide polyoxometalates	Cell-free system and cellular model of AD	↑SOD-like activity, ↓cellular ROS	-	↑cell viability under Aβ cytotoxicity	↑degradation of Aβ monomers and disaggregation of Aβ fibrils, ↓Aβ aggregation	↓microglial activation	[353]
	Nanorods of CeVO_4_	Cellular model of ALS	↑SOD-like activity, ↓cellular and mitochondrial ROS	↑ATP levels, ↑stability of mitochondrial membranes	↑cell viability and ↓apoptotic pathway	-	-	[354]
	MoS_2_-TPP	Cellular and mouse models of AD	↑Activity of antioxidant-like enzymes, ↓cellular and mitochondrial ROS	↑mitochondrial localization, ↓structural alteration and degradation of mitochondria, ↑mitophagy	↑levels of neuroprotective protein NEuN	↓Aβ aggregation and deposition in hippocampus	↑permeation through a BBB model, ↑microglial transition from pro- inflammatory M1 to anti- inflammatory M2 state	[355]
	Cu*x*O NPs	Cellular and mouse models of PD	↑Activity of antioxidant-like enzymes, ↓ROS and lipid oxidation levels	-	↑cell viability in PD cells model, ↑viability of dopaminergic neurons in PD mice, ↓apoptotic pathway	-	↑dopamine levels, ↑memory function	[356]
Carbon-based nanozymes	GQD and GOQD	Cellular and animal models of PD	↓ROS levels, ↑activity of antioxidant-like enzymes	↑stability of mitochondrial structure in brain cells	↓dopaminergic neuron loss, ↓apoptotic pathway	↓α-syn and Aβ aggregation, ↑dissociation of α-syn fibrils	↑locomotor function	[294, 357]
	Carboxyfullerene (C60)	Cellular neuroinflammatory and animal models of PD and HD	↓ROS levels, ↑ROS scavenging and GSH levels in mitochondria from HD rats	↑Activity of ETC complexes, ↑stability of mitochondrial membranes, ↓mitochondrial fragmentation and DRP1 activity	↑stability and preservation of dopaminergic neurons, ↓apoptotic pathway	-	↓inflammatory markers, ↑mouse lifespan, ↑dopamine levels and motor function in a PD primate model	[358,359,360]

GO: graphene oxide, GQD: graphene quantum dots, GOQD: graphene oxide quantum dots, MoS2-TPP: molybdenum disulfide quantum dots associated to triphenylphosphonium, CuxO NPs: nanoparticles of Cu (II) ions coordinated to phenylalanine structure, ROS: reactive oxygen species, Aβ: amyloid β, DRP1: dynamin-related protein, α-syn: α-synuclein, ETC: electron transport chain, mPTP: mitochondrial permeability transition pore, GSH: glutathione, PD: Parkinson’s disease, AD: Alzheimer’s disease, ALS: amyotrophic lateral sclerosis, HD: Huntington’s disease, MS: multiple sclerosis.

## Data Availability

Not applicable.
